# Leptomeningeal Dissemination Complicated With Acute Tetraplegia From a Supratentorial Multicentric Isocitrate Dehydrogenase (IDH)-Wildtype Glioblastoma: A Case Report

**DOI:** 10.7759/cureus.55777

**Published:** 2024-03-08

**Authors:** Dan M Visarion, Ionut Cale, Ioana Miron, Bogdan I David, George E Petrescu, Viorel M Pruna

**Affiliations:** 1 Neurosurgery Department, Carol Davila University of Medicine and Pharmacy, Bucharest, ROU; 2 Neurosurgery, Bagdasar-Arseni Emergency Clinical Hospital, Bucharest, ROU

**Keywords:** : multiple spinal metastases, aseptic meningitis, tetraplegia, leptomeningeal carcinomatosis (lmc) cerebrospinal fluid (csf), leptomeningeal dissemination, idh-wildtype glioblastoma, hydrocephalus

## Abstract

Glioblastoma (GBM) is a major concern for neurosurgeons and oncologists, being a malignant tumor with a high recurrence rate and reduced survival. Leptomeningeal dissemination (LMD) of GBM is rare and difficult to diagnose due to the low rate of cellular detection in the cerebrospinal fluid and clinical and imaging similarities with fungal and tuberculous meningitis. We report the case of a 25-year-old female patient suffering from multicentric GBM who developed hydrocephalus and extensive LMD three months after surgery for a left frontal parafalcine cerebral GBM isocitrate dehydrogenase (IDH)-wildtype.

## Introduction

Primary malignant central nervous system (CNS) tumors represent 2% of all types of cancer, of which more than 50% are glioblastomas (GBMs) [1,2]. The leptomeningeal dissemination (LMD) of GBM is poorly documented, and insufficient data exist to evaluate its incidence and prevalence [3]. A few studies suggest that there may be multiple risk factors for dissemination, including surgical resection, surgical opening of the ventricles, ventricular invasion, and immunosuppression [4].

## Case presentation

**Figure 1:**
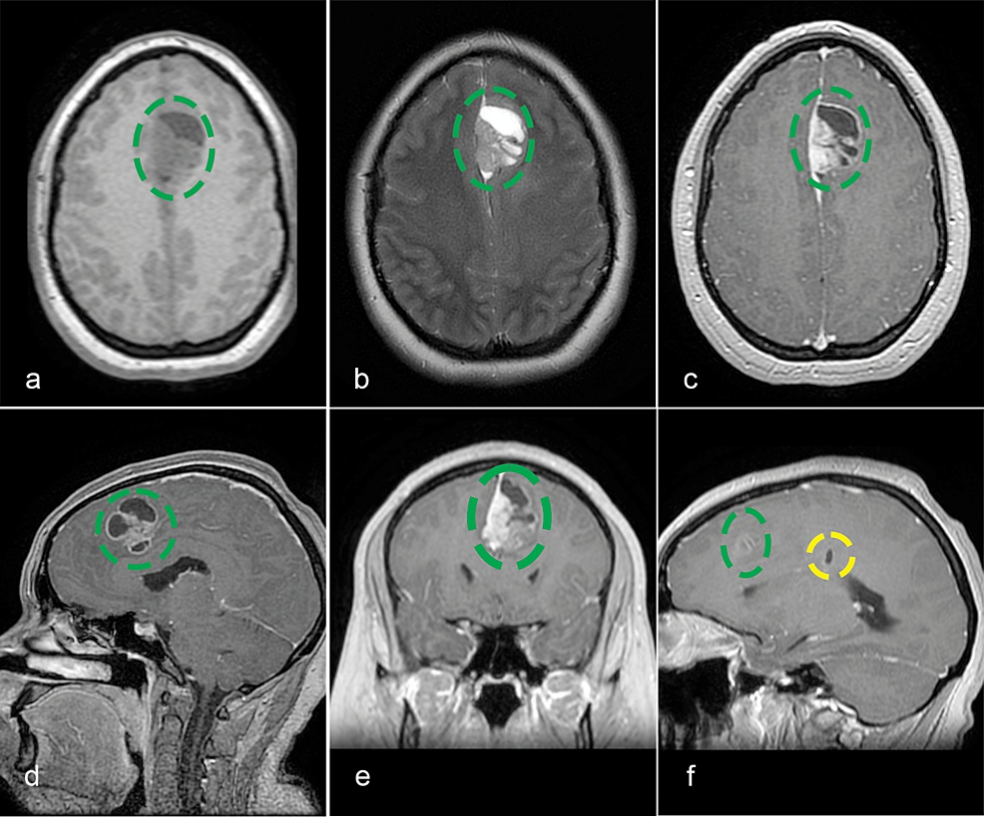
Enhanced MRI shows a left frontal parafalcine cerebral tumor (green circle). a: axial T1; b: axial T2; c: axial contrast-enhanced T1; d: sagittal contrast-enhanced T1; e: coronal contrast-enhanced T1; f: sagittal contrast-enhanced T1 depicting a small cystic lesion (yellow circle) in the left parietal lobe next to the lateral ventricle

A 25-year-old female patient presented in February 2023 with persistent headaches and frontal lobe syndrome. Contrast-enhanced MRI revealed a left frontal parafalcine cerebral tumor and one small cystic lesion in the left parietal lobe near the lateral ventricle (Figures 1a-1f). She underwent a gross total resection of the parafalcine frontal lesion, but due to intraoperative cerebral edema, the bone flap could not be placed. After the surgery, the patient remained neurologically intact. However, a CT scan revealed a parietal extradural hematoma, prompting a second surgery for hematoma evacuation. Afterwards, recovery was uneventful; the patient was discharged with a modified Rankin Score (mRS) of 1 and referred for oncological treatment. Due to its cortical, almost dural-based position, anaplastic pleomorphic xanthoastrocytoma was suspected. Histopathological examination confirmed our supposition but could not exclude a GBM. Immunohistochemical examination revealed isocitrate dehydrogenase (IDH)-wildtype, CNS WHO grade 4 GBM (Table 1).﻿

**Table 1:** Imunohistochemistry tests reveal IDH-wildtype, CNS WHO grade 4 glioblastoma

Immunohistochemistry tests	Result
IDH1	Negative
ATRX	Diffuse nuclear intense positivity
OLIG2	Intense nuclear expression in 60% of tumoral cells
KI-67	Intense positivity in 45% of tumoral cells
GFAP	Moderate and intense expression – heterogenic
SYNAPTOPHYSIN	Heterogenic expression
CD56	Diffuse intense expression
P53	Intense nuclear positivity – wild type pattern, non-mutant phenotype

**Figure 2:**
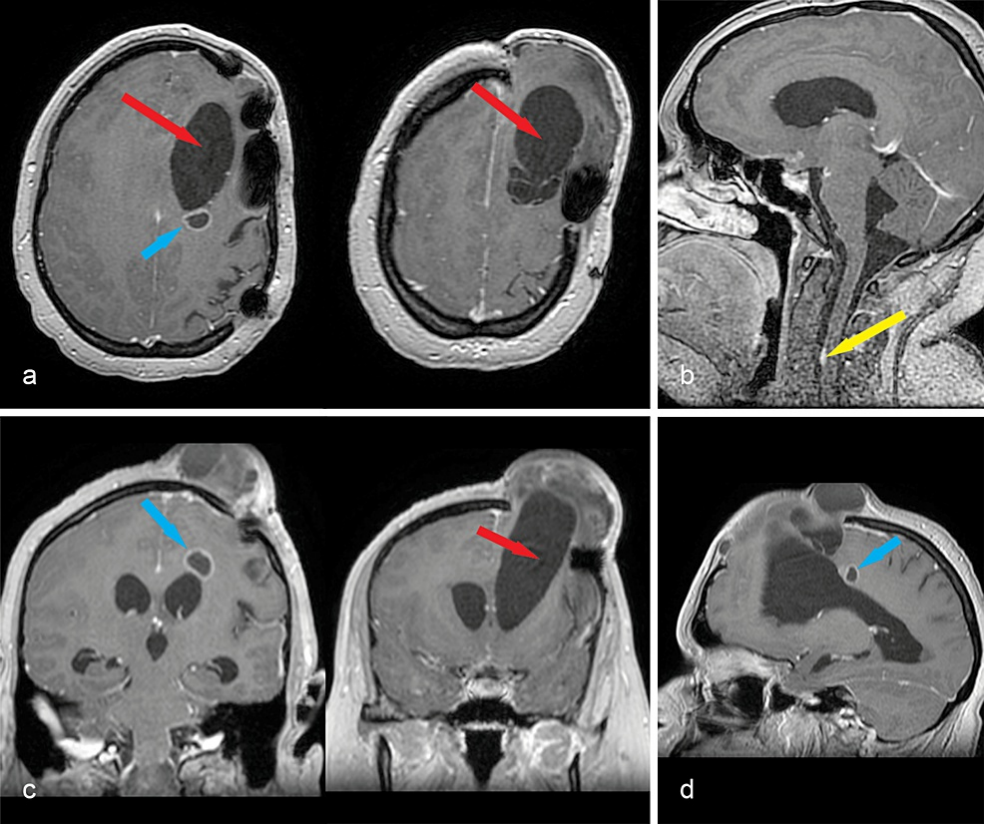
a, c, d: axial, coronal, and sagittal contrast-enhanced T1 MRI sequences depict the cystic mass (red arrow) causing retraction and dilation of the left frontal horn, with possible communication with the ventricle; the cystic lesion and the ventricular horn prolapsed through the bone defect; the ventricles are enlarged, with periventricular resorption halo; the second, parietal tumor is slightly enlarged (blue arrow); b: sagittal contrast-enhanced T1 sequence shows the new dural-based lesion next to the C3 vertebra (yellow arrow)

The patient underwent standard oncological treatment, including whole-brain radiation therapy (WBRT) (31 out of 33 recommended sessions) and temozolomide 75 mg/m2 for 42 days. In May 2023, during the 31st radiotherapy session, the patient experienced gait disturbances and progressive expansion through the bone defect. Contrast-enhanced MRI revealed a left frontal parasagittal multicystic mass and dilated ventricular system (Figures 2a, 2c, 2d). The small parietal paraventricular tumor was slightly enlarged. Moreover, a new, small gadolinophilic lesion was observed on the anterior dura adjacent to the C3 vertebral body (Figure 2b).

Afterwards, a lumbar puncture was performed, and a high cerebrospinal fluid (CSF) opening pressure was noted. The basic biochemical evaluation was unremarkable (three elements, Pandy's reaction positive ++++, subtle xanthochromia). The expansion of the frontal cavity decreased, so we decided to treat the patient for secondary hydrocephalus. A unishunt ventriculoperitoneal drainage was placed into the right lateral ventricle using the Keen point. Intraventricular pressure was 450 mmH2O, and the biochemical assay showed clear CSF, 2 elements, and a positive Pandy's reaction of ++++ (meaning >1 g/1000, according to our laboratory reference). Following surgery, the frontal breach collapsed completely. Over the next two weeks, the drainage system presented multiple malfunctions, leading to re-expansion of the frontal breach. Consequently, the patient underwent three additional surgeries. Each time, a basic CSF biochemical exam was performed, revealing a protein level >7 g/1000, subtle xanthochromia, and 5-8 cells in the CSF cell count.

**Figure 3:**
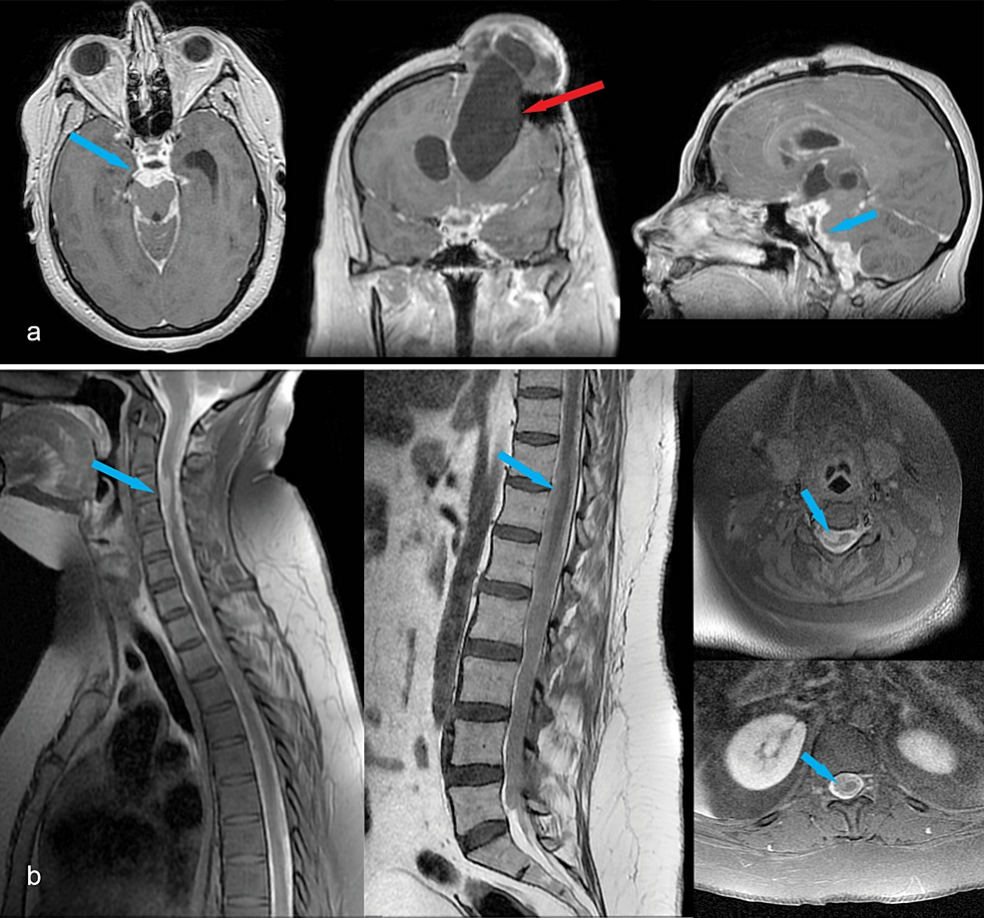
a: axial, coronal, and sagittal contrast-enhanced T1 MRI show the right ventricle collapsed, the left ventricle dilated and deformed (red arrow); basal and infratentorial cisterns, cranial nerves, and both sylvian fissures present pseudo-nodular, contrast-enhancing thickenings (blue arrows); b: sagittal and axial spinal T2 MRI depicts similar lesions throughout the entire spinal dura mater (blue arrows)

Due to the challenging clinical course, we performed cerebral and whole spine enhanced MRI, which revealed contrast enhancement of the cystic lesions, a collapsed right lateral ventricle, and a dilated and deformed left ventricle (Figure 3a), with extensive leptomeningeal pseudo-nodular thickening (Figure 3b).﻿

**Figure 4:**
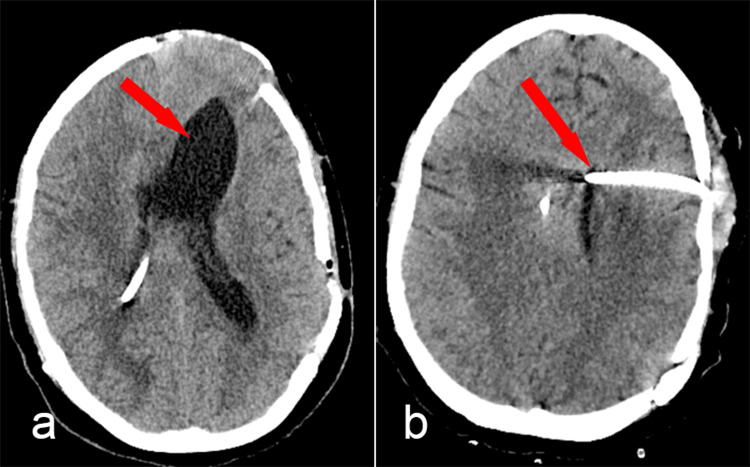
a: CT scan showing functional right ventricle catheter and dilated left ventricle (red arrow); b: CT scan after the insertion of the second catheter into the left ventricle (red arrow)

**Figure 5:**
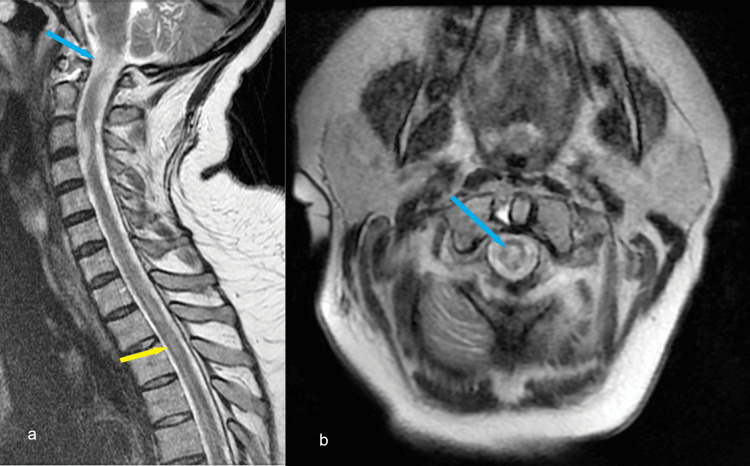
a: sagittal T2 MRI showing new C1-C2 (blue arrow) and T2-T3 intramedullary T2 hyperintense areas (yellow arrow); b: axial T2 sequence of the C1-C2 intramedullary lesion (blue arrow)

The patient became somnolent and suffered partial right eyelid ptosis. Due to the significant difference in size between the right and left lateral ventricles (Figure 4a), we decided to place an external ventricular drainage on the left side (Figure 4b). The surgery was uneventful, and CSF samples were collected to evaluate a possible meningitis. However, immediately after the surgery, the patient woke up tetraplegic. A cervical MRI was performed immediately after surgery, revealing two new acute intramedullary hyperintense lesions on the T2 sequence with minimal restricted diffusion and no enlargement of the spinal cord (Figures 5a, 5b). Due to extensive dural pseudo-nodular thickening, meningitis was our first suspicion. CSF had mild cellularity, so bacterial meningitis was considered less likely. A full panel of CSF examinations was performed (Table 2).﻿

**Table 2:** Full CSF panel examination, including Cryptococcus neoformans, Mycobacterium tuberculosis, and cytologic examination

CSF analysis	Result
Pandy’s reaction	++++
CSF nucleated cells count	6/mmc
Giemsa stain	rare mononuclear cells, very frequent red blood cells
Gram stain	round, in diplo gram-positive cocci: absent; round, in diplo gram-negative cocci: absent; gram-negative bacilli and coccobacilli: absent; short, thin, gram-positive bacilli: absent; oval formations (yeast fungi): absent
Bacterial culture	*Staphylococcus spp., Streptococcus spp., Enterococcus spp., Enterobacterales, Pseudomonas spp., Acinetobacter spp., Haemophilus spp., Neisseria meningitidis, Listeria monocytogenes*: absent
India ink stain (*Cryptococcus neoformans*)	negative
Mycological culture	negative after 5 days
Ziehl-Neelsen stain	negative
*Mycobacterium tuberculosis* culture in Lowenstein-Jensen medium	negative after 60 days

**Figure 6:**
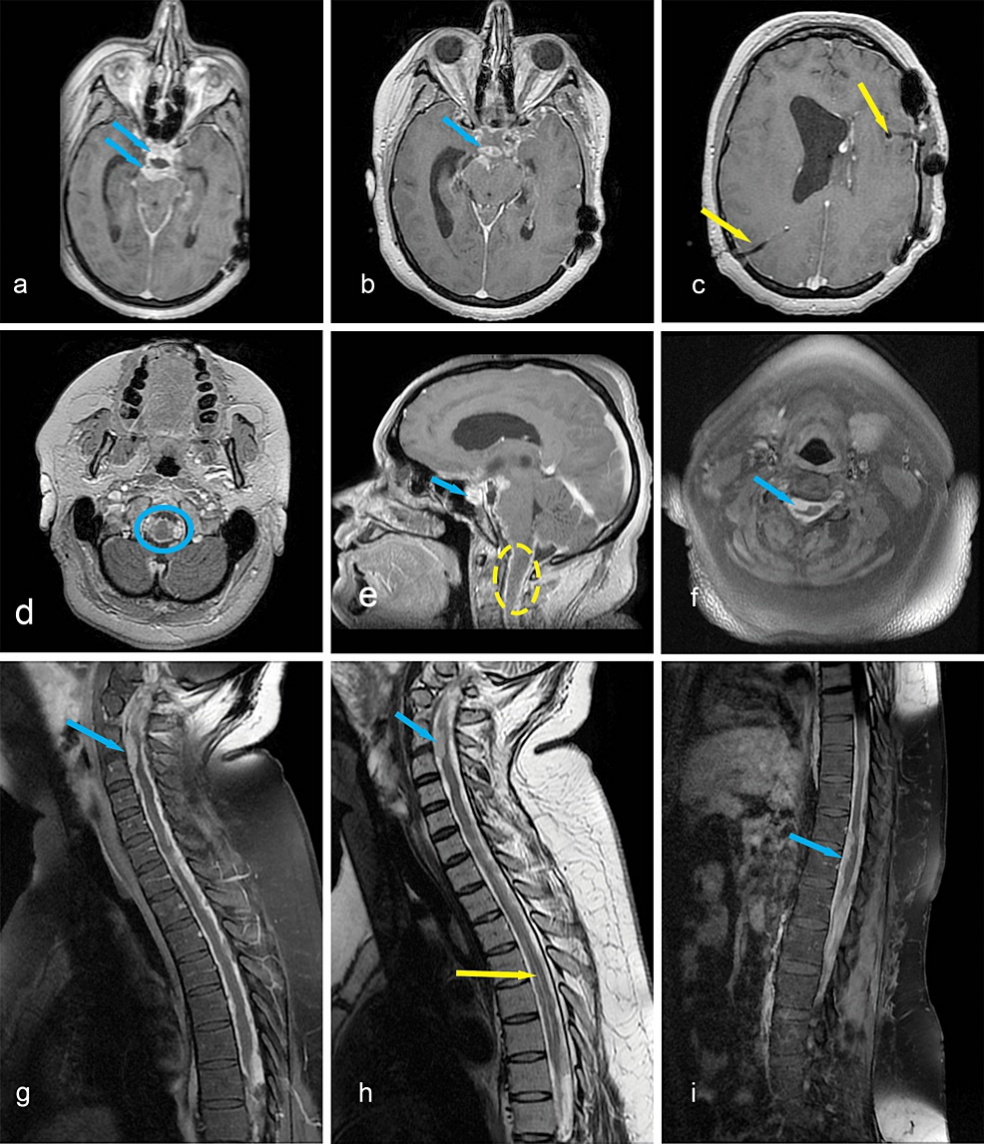
a, b, d: axial contrast-enhanced T1 cerebral MRI depicting leptomeningeal lesions (blue arrows, blue circle); c: axial contrast-enhanced T1 showing the catheters in both lateral ventricles (yellow arrows); e: sagittal contrast-enhanced T1 depicting extensie leptomeningeal enhancement (blue arrow), and the C1/C2 intramedullary lesion persisted, without expansion of the spinal cord, nor contrast enhancement (yellow circle); f: axial contrast-enhanced T1 showing the C3 dural lesion (blue arrow); g, h: contrast-enhanced T1 and non-enhanced T2 cervicothoracic MR image revealing discrete progression, extensive dural thickening (blue arrows), and dilated ependymal canal (yellow arrow); i: thoracolumbar contrast-enhanced T1 image showing extensie dural thickening (blue arrow)

Antifungal and broad-spectrum antibacterial therapy was initiated, along with methylprednisolone, while awaiting the results. Methylprednisolone was started immediately after the C1-C2 intramedullary lesion appeared on the MRI to cover possible causes of transverse myelitis. The patient temporarily regained mobility in the left arm and had some voluntary contractions in the left leg. Two days later, another contrast-enhanced cerebral and spinal MRI was performed, showing discrete dimensional progression of the dural pseudo-nodular lesions and enlargement of the ependymal canal (Figure 6a-6i). The left external ventricular drain was later connected to the right ventriculoperitoneal drain using a Y-shaped connector. Another CSF sample was analyzed, and the results showed 50 cells at the CSF cell count (out of which 60% were polymorphonuclear neutrophils) and lower protein levels (Pandy’s reaction ++). ﻿

Five days after the last surgery, the patient suffered neurologic deterioration complicated by pneumonia, necessitating intubation and mechanical ventilation. Subsequently, she suffered two cardiac arrests within the next five days, and the last one could not be resuscitated.

## Discussion

Despite GBM’s high rate of local recurrence, LMD and spinal metastases are exceedingly rare. According to the literature, dissemination occurs in younger patients [5]. LMD can mimic infectious/inflammatory meningitis, while the focal form, depicting spinal pseudo-tumoral lesions, can mimic dural-based tumors [6,7]. Spinal metastases are usually multifocal, multiplanar lesions displaying a predilection for the thoracic region, followed by the lumbar and cervical segments [3].

In a study of 15 patients with GBM and spinal metastases, the interval between the diagnosis of GBM and the identification of LMD ranged between 1 and 49 months, while survival varied from 1 to 14 months [8]. Arita et al. presented similar outcomes in patients with LMD, with survival varying from 2 to 39 weeks (mean 19 weeks) [9]. Ammerman et al. presented a case of acute tetraplegia and cardiac arrest from a cervical leptomeningeal metastasis in less than five months after the resection of a giant cell GBM [10]. Clinical evaluation of patients with LMD requires a differential diagnosis between bacterial, fungal, and carcinomatous meningitis. In immunosuppressed patients, the first hypothesis is infectious meningitis rather than a carcinomatous one. In our case, multiple lumbar punctures were performed, and ventricular CSF was obtained via a ventricular catheter. The CSF was analyzed repeatedly, revealing hyperproteinemia and monocytes, but no tumoral cells were found. CSF analysis has a low rate of detecting tumor cells, but the sensitivity may be increased by almost 90% by consecutive lumbar punctures (more than three) [7,11]. Considering that in our case, monocytes were found in the CSF, it is important to mention that the differential diagnosis between monocytes and GBM tumoral cells is difficult to make via standard laboratory examinations [12]. We performed two Giemsa stains, one in our histopathological department and one at a hospital for infectious diseases, and both revealed the same results. Extensive microbiologic work-up was needed due to similarities with some infections, such as cryptococcal infection (monocytes, hyperproteinemia, and normal glycorrhachia) or tuberculous meningitis (hyperproteinemia, monocytes, and low CSF glucose levels) [5,7,13,14].

Studies have found two different patterns in the progression of GBM: the most common pattern is characterized by local tumor growth/recurrence, while the second pattern is characterized by aggressive CSF dissemination (cranial and spinal leptomeningeal metastases) and a lower rate of local tumor growth/recurrence [5,7,15]. Our patient exhibited the second pattern, with minimal local tumor growth but violent leptomeningeal and spinal dissemination. The incidence of GBM dissemination remains underestimated because the local evolution of these tumors is very aggressive; therefore, metastases remain asymptomatic and underdiagnosed [5,7]. Many studies, including autopsies, showed a rate of up to 25% of GBM leptomeningeal metastases [7,11,16].

The clinical presentation of patients with LMD varies from asymptomatic to severely symptomatic [7,11,16]. In most cases, symptoms evolved progressively, but the sudden onset of symptoms was also noted. Symptoms include increased intracranial pressure, cranial nerve palsies, focal neurological deficits, hydrocephalus, progressive paraplegia, and spinal ataxia [7,10]. Communicating hydrocephalus is diagnosed in 25-40% of patients with LMD [7,17,18]. Moreover, shortly after admission, the patient experienced multiple generalized seizures and decreased cognitive function, which is common among patients with LMD. According to the literature, dissemination occurs through the CSF and is favored by the presence of several risk factors, such as midline location, histological grade, surgical resection, surgical opening of the ventricles, ventricular invasion, and decreased immune function [4,5]. Roelz et al. found a statistically significant correlation in univariate analysis between how close the tumor was to the ventricles and the risk of CSF dissemination [14]. In our case, the midline location of the resected lesion and the periventricular disposition of the second, smaller tumor were the only risk factors for CSF dissemination.

GBM with leptomeningeal spread was suspected based on the correlation of the typical imaging pattern and microbiological examination of CSF, which revealed no germs but only high protein levels and inflammatory cells on multiple CSF analyses. The patient did not improve under methylprednisolone (to cover a possible cause of tetraplegia from transverse myelitis) [19], antifungal, or broad-spectrum antibiotic therapy. There is no established treatment, and actual recommendations involve radio-chemotherapy as well as surgical intervention for focal metastatic lesions [3,8]. Due to the rapid, unfavorable course of the disease, palliative treatment could not be performed.

## Conclusions

GBM LMD is rare, and differential diagnosis between infectious and carcinomatous meningitis can be difficult, especially when no tumor cells are found in the CSF. To increase the sensitivity of CSF cytology, multiple CSF samples should be analyzed. The clinical course can be fulminant, with multiple complications along the way and a final unfavorable outcome. In GBM patients with suggestive imaging features, LMD should be considered after excluding common infectious pathogens.
